# Intracranial Ischemic Infarct Due to Blunt Force Trauma in a High School Football Player

**DOI:** 10.7759/cureus.1659

**Published:** 2017-09-07

**Authors:** Brandon I Esianor, Ali S Haider, Margaret I Engelhardt, Tijani Osumah, Steven Vayalumkal, Richa Thakur, Dean Leonard, Jeffrey Haithcock, Kennith F Layton

**Affiliations:** 1 Otolaryngology, Mcgovern Medical School; 2 Neurosurgery, Texas A&M College of Medicine; 3 School of Medicine, Ross University; 4 School of Medicine, St. George's University; 5 Texas A&M College of Medicine; 6 Department of Radiology, Baylor University Medical Center

**Keywords:** internal carotid artery dissection, head trauma, traumatic brain injury (tbi), stroke, athlete, sports trauma

## Abstract

Ischemic stroke is an uncommon cause of death among teenagers and young adults; however, the etiologies differ when compared to ischemic strokes in older individuals. Large-vessel atherosclerosis and small-vessel disease causing ischemic stroke are rare for the teenage population, while cervicocerebral arterial dissections account for up to 20% of ischemic strokes. Here, we present the case of a 16-year-old male who developed internal carotid artery dissection (ICAD) after a head injury and subsequently developed ischemic stroke and seizures.

## Introduction

Ischemic stroke is a relatively uncommon cause of morbidity and mortality in teenagers and young adults, with an annual occurrence rate of 10.8 per 100,000 people aged 15-49 years and exponential risk dependent on age [1]. In teenagers aged 15-19 years, typical adult etiologies, such as large-artery atherosclerosis and small-vessel disease, are extremely rare, while cervicocerebral arterial dissections are an over-represented etiology, accounting for up to 15% of young strokes [1]. Traumatic whiplash injuries are a known cause of internal carotid artery dissections (ICAD), particularly those involving rotation-hyperextension or distraction-flexion forces. Traumatic ICAD typically presents with a brief interval without immediate symptoms followed by hemiplegia, hemianesthesia, unilateral facial weakness, aphasia, amaurosis fugax, ipsilateral Horner’s syndrome, and seizures usually within 24 hours [2]. Extracranial traumatic ICAD involving the cervical portion is the most common location, with intracranial traumatic dissections considered much more rare, with few reported cases in children [3-4]. Traumatic brain injuries can also induce post-traumatic vasospasm, with a milder course, earlier onset, and shorter duration than vasospasm induced by aneurysmal subarachnoid hemorrhage (SAH) [5]. Here, we present an interesting case of a young male with a right middle cerebral artery (MCA) ischemic infarct secondary to blunt force trauma to the head with likely intracranial ICAD.

## Case presentation

**Figure 1:**
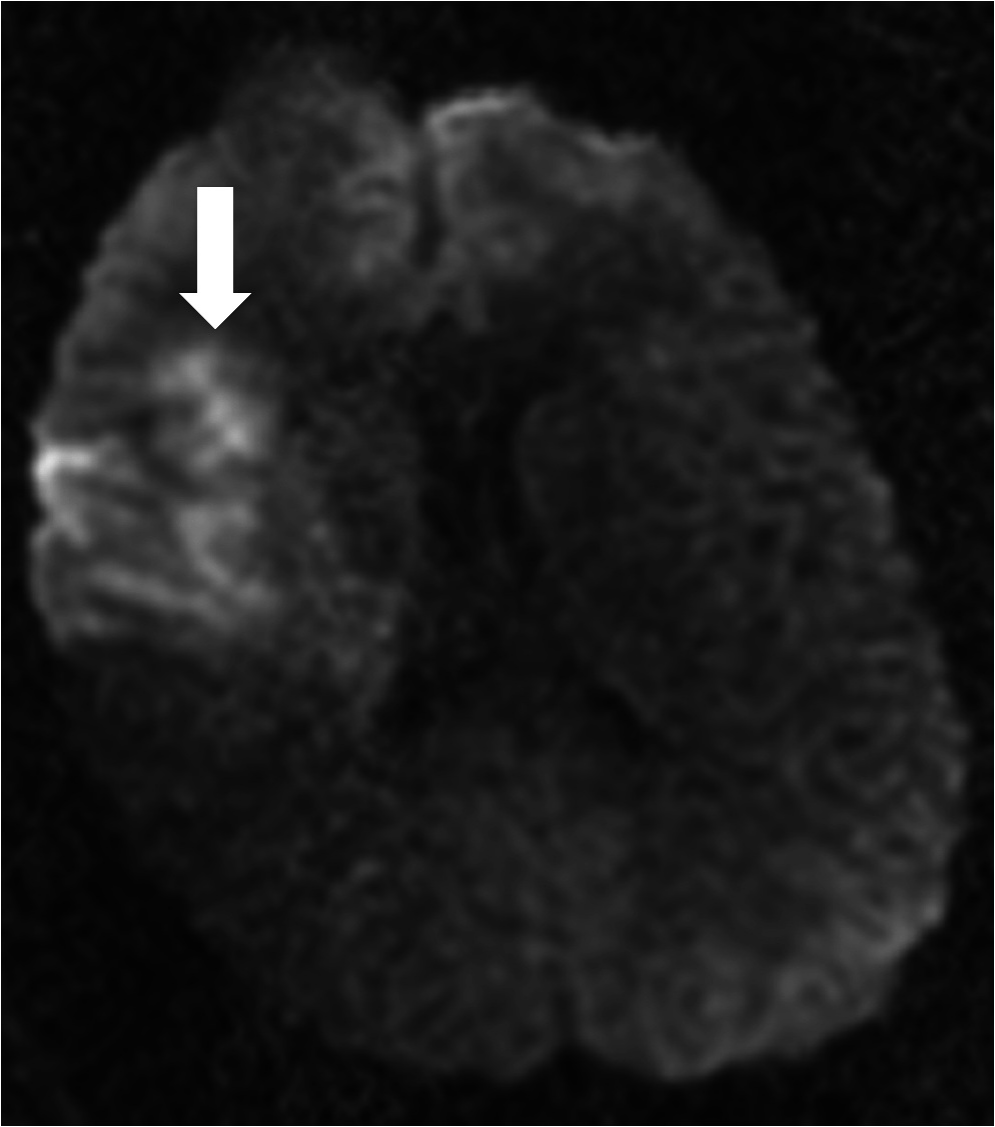
Diffusion-weighted image from a brain magnetic resonance image on the day of presentation reveals an acute infarct in the right middle cerebral artery territory.

A 16-year-old male with no previous medical history experienced a transient loss of consciousness after two simultaneous head-to-head collisions during a high school football game. Upon regaining consciousness, the patient initially answered questions appropriately; however, he became combative and was intubated due to severe agitation and suspected seizure activity. The patient was transferred to an outside hospital and received computed tomography (CT) scans of the head and cervical spine, both of which reported no acute pathology. An attempt was made to extubate the patient; however, a neurological examination revealed 3/5 left-sided strength with spasticity. Sensation was intact bilaterally, and no carotid bruits were heard. Brain and cervical spine magnetic resonance imaging (MRI) revealed a right MCA infarct on the diffusion-weighted image and questionable right internal carotid artery (ICA) dissection (Figure 1).

The patient was started on heparin and a CT angiography revealed mild irregularity along with segmental narrowing of the distal cavernous and supraclinoid segments of the right internal carotid artery.

**Figure 2:**
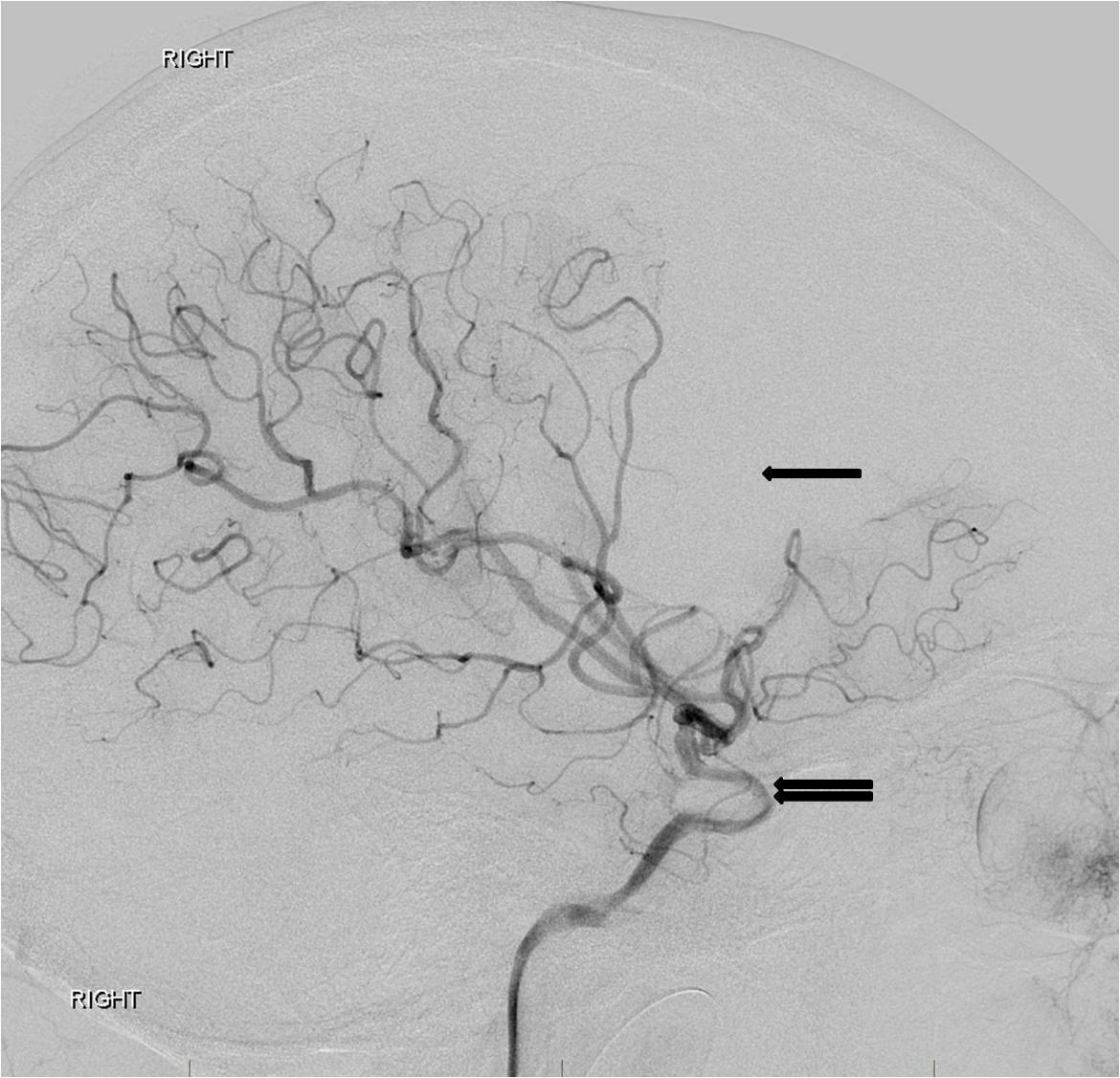
Lateral digital subtraction angiography image from a right internal carotid artery angiogram reveals a narrow and irregular supraclinoid internal carotid artery (double arrow) with distal middle cerebral artery branch occlusions as a wedge-shaped area of absent perfusion (single arrow). The appearance is consistent with a traumatic dissection of the supraclinoid right internal carotid artery with associated distal right middle cerebral artery emboli. "Right" indicates the patient's right side.

**Figure 3:**
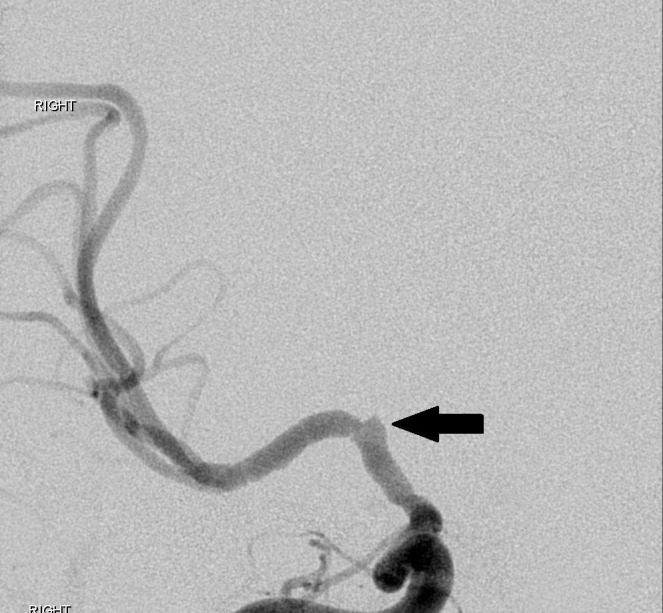
Cerebral angiogram image demonstrating irregularity of the supraclinoid segment of the right internal carotid artery (arrow) and the complete absence of lenticulostriate vessels arising from the M1 segment of the right middle cerebral artery. "Right" indicates the patient's right side.

**Figure 4:**
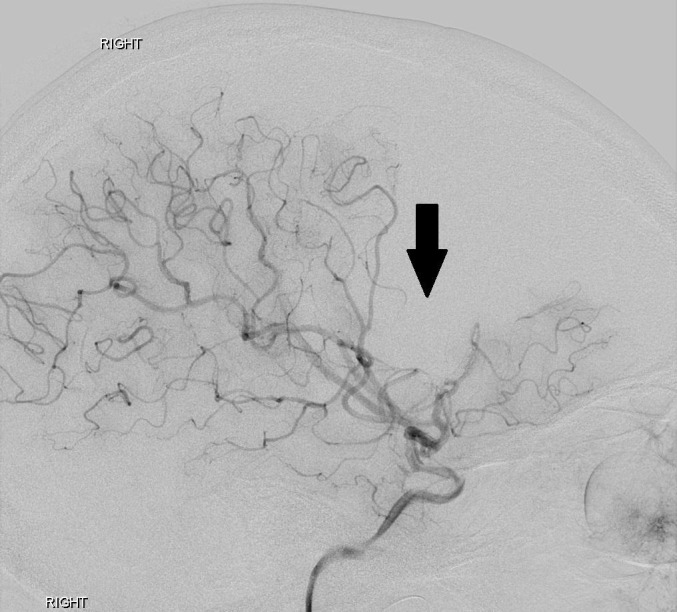
Cerebral angiogram image demonstrating the area of ischemic injury secondary to distal thromboembolic occlusion. "Right" indicates the patient's right side.

Upon arrival to our institution, sedation was turned off and the patient was noted to have 1/5 left extremity strength on repeat neurological exam. Neurology and neurosurgery were consulted and the decision was made to perform a cerebral angiogram. The study showed multifocal irregularities of the right supraclinoid internal carotid artery and the M1 segment of the right MCA, highly suggestive of nonflow limiting vasospasm. Distal emboli were observed in the right MCA (Figures 2, 3, 4).

**Figure 5:**
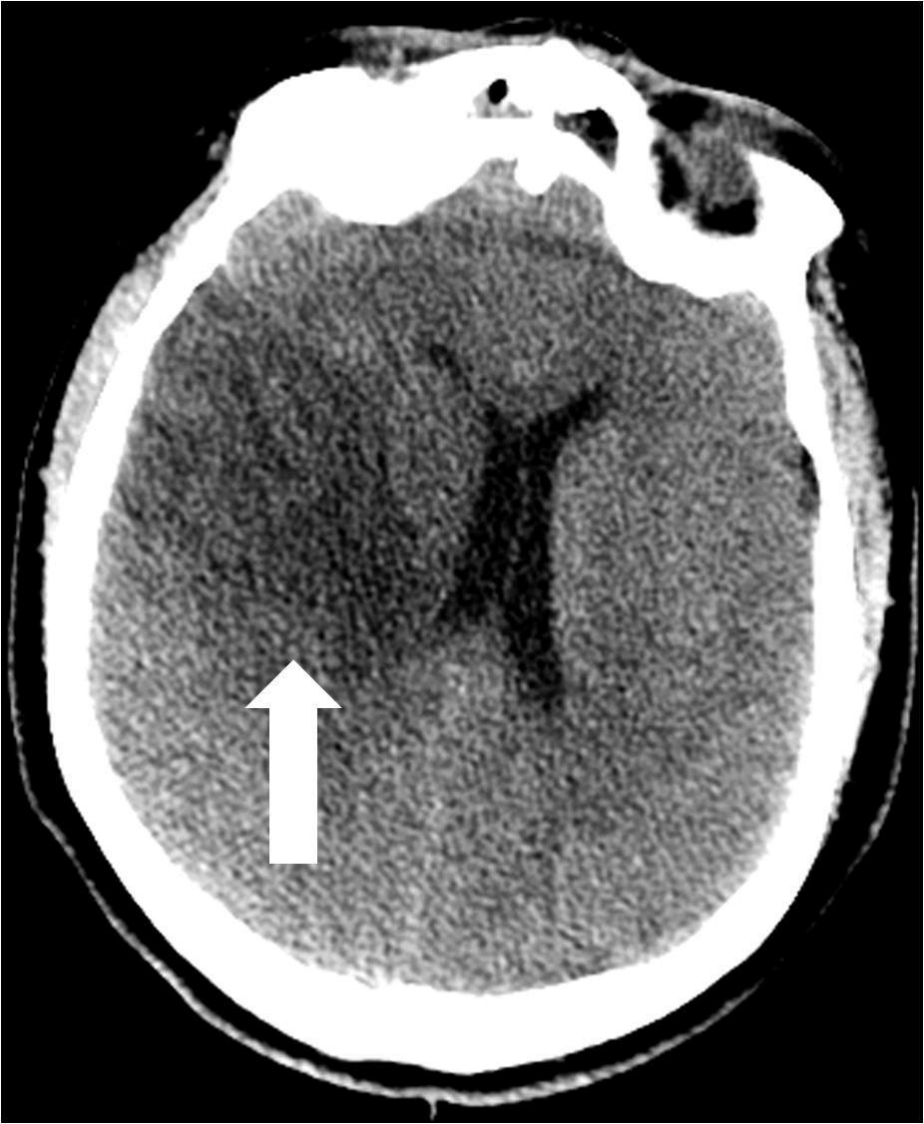
Computed tomography of the head performed five days after the traumatic event reveals the expected evolution of the right middle cerebral artery distribution infarct (arrow) with mild mass effect and right-to-left midline shift.

An electroencephalogram (EEG) was performed and the presence of a mild encephalopathy with superimposed right temporal slowing was noted. No epileptiform activity was seen. The patient was started on hypertonic saline due to cerebral edema, valproic acid, and aspirin 325 mg. He was extubated one day after arrival at our facility. Serial head CT scans were performed and revealed progressive ischemic changes involving territories of the right MCA, anterior cerebral artery (ACA), anterior choroidal artery, and medial ventriculostriate perforator arteries and a stable 6 mm midline shift (Figure 5).

No extension of the infarct was noted. Transcranial Doppler ultrasonography (TCD) showed significantly elevated velocity in the right MCA. However, hypertonic saline was appropriately tapered and significant improvement was noted on repeat TCD. The patient was transitioned from valproic acid to levetiracetam and discharged to inpatient rehab for continued care. At the time of discharge, a neurological examination revealed 0/5 strength in the left upper and lower extremities, with left pronator drift and upgoing left plantar reflex.

**Figure 6:**
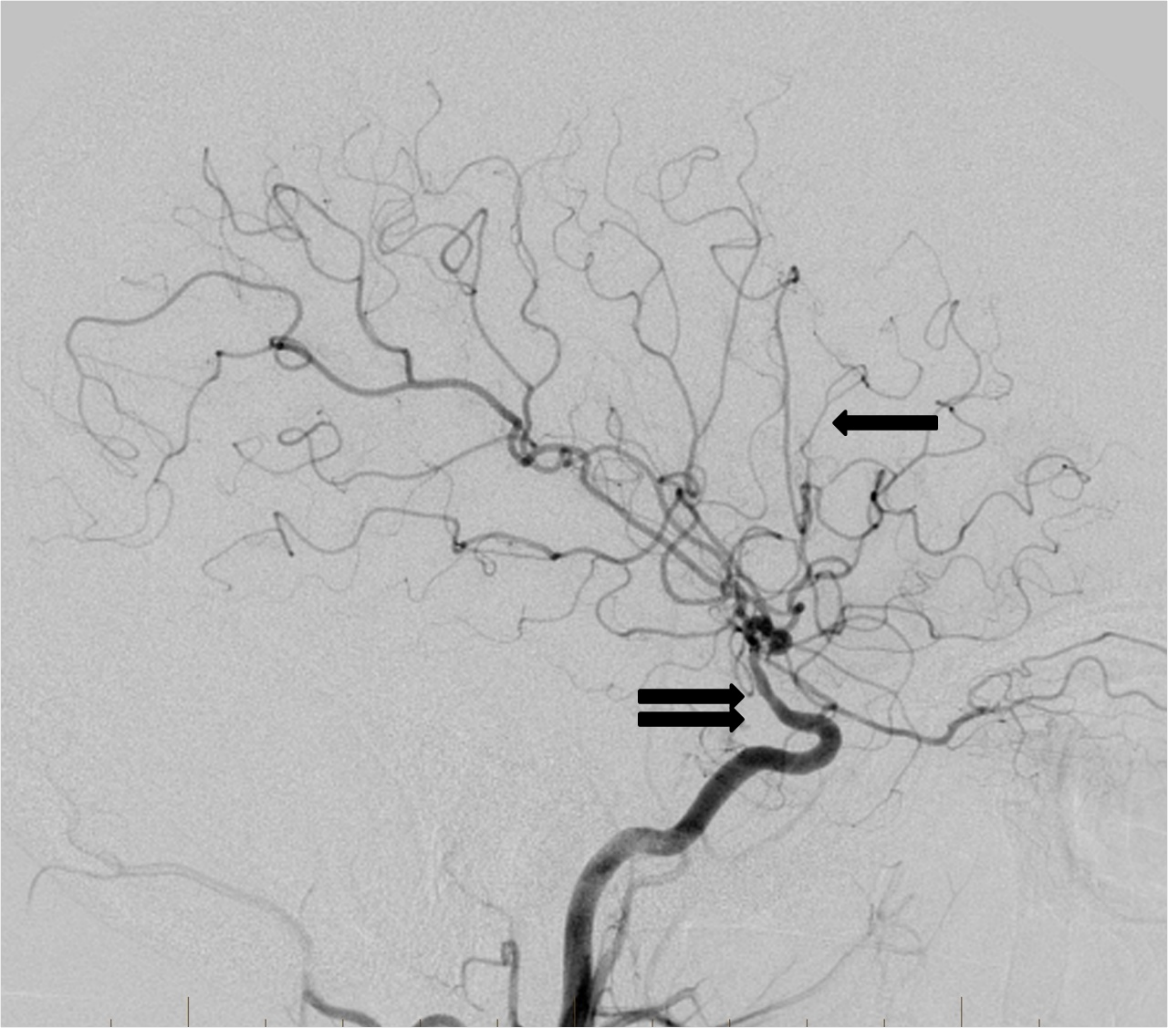
Follow-up cerebral angiogram lateral projection from a right internal carotid artery injection three months after the traumatic event reveals persistent abnormal narrowing of the supraclinoid internal carotid artery (double arrow). The previously occluded distal right middle cerebral artery branches have recanalized (single arrow). Importantly, no pseudoaneurysm development has occurred.

A follow-up cerebral angiogram was performed four months after the initial injury, which revealed progressive segmental luminal irregularity of the right supraclinoid internal carotid artery extending into the right M1 segment, as well as globally diminished caliber of the right MCA territory branches. These findings were unchanged from a CT angiogram performed one month earlier (Figure 6). At the time of the follow-up angiogram, a neurological examination revealed 4/5 strength in the left upper and lower extremities. 

## Discussion

This case involves a young patient with ischemic injury due to possible right ICA dissection involving the right cavernous and supraclinoid segments versus vasospasm. The involvement of multiple anterior circulation branches from the ICA, particularly the involvement of the anterior choroidal artery and the medial lenticulostriate perforator arteries, is suggestive of traumatic ICA dissection. Ischemic injury secondary to ICAD results from stenotic flow through a dissected vessel or embolic arterial occlusion secondary to thrombus formation. The majority of infarcts after ICAD occur at the site of the vascular injury [6]. Several clinical features separate extracranial and intracranial ICAD, with intracranial ICAD more likely to (1) affect younger patients, (2) be subintimal in location, as opposed to involving the media or adventitia, and (3) precipitate large-volume infarcts and rapid onset of neurological deficits [3,7]. Conventional intra-arterial angiography continues to be the gold standard for the diagnosis of ICAD, regardless of location, with magnetic resonance angiography (MRA) being the preferred noninvasive technique [4].

Transcranial Doppler has been used to demonstrate that hemodynamically significant vasospasm frequently occurs after traumatic brain injury (TBI), as shown by increased flow rates through the constricted vessels. In patients with severe blunt head injury, up to 40% experience MCA spasms, as demonstrated on TCD; flow rates reach a maximum at five to seven days after injury and usually resolve within 14 days [8]. This reaction can occur even in the absence of intracranial bleeding, such as traumatic SAH [5]. The development of vasospasm after TBI is correlated with younger age and lower Glasgow Coma Scale (GCS) scores on admission [9].

In our case, the patient displayed transiently elevated velocity in the right MCA without signs of intracranial hemorrhage on imaging, consistent with post-traumatic vasospasm. However, it is unlikely that non-SAH-related vasospasm alone could account for the degree of MCA infarct and subsequent neurological impairment. Instead, post-traumatic vasospasm is likely a compounding secondary insult seen in traumatic ICAD resulting from the underlying brain injury.

## Conclusions

Traumatic brain injury in high school athletes is an increasingly prevalent and devastating cause of morbidity and mortality. Head injuries can lead to ischemic stroke secondary to internal carotid artery dissection. Further studies and evidence-based guidelines are needed to best prevent these potentially fatal injuries in high school sporting events.
